# News in Brief

**Published:** 1922-03

**Authors:** 


### NEWS IN BRIEF.

### Personal Notes—What the Hospitals are Doing.

Dr. Herbert Keighley has been re-elected president of Batley Hospital.

The first convalescent home of the Teachers' Provident Society has been opened in Henry Avenue, Matlock.

Mr. A. H. Ough, honorary secretary to the Dawlish Hospital Committee, has resigned after some differences of opinion.

The League of the Roses (Miss M. F. Roby, chairman) contributed £1,900 to the Royal Northern Hospital last year.

Though the Sheffield Royal Hospital has decided not to close wards at present, its overdraft amounts to some £55,000.

Sir Robert Jones, K.B.E., C.B., F.R.C.S., has consented to become the President of the Association of Certificated Blind Masseurs.

Mr. J. H. Stainton is editing a volume on "The Making of Sheffield," to be issued in four parts at 15s. each for the benefit of the Sheffield hospitals.

St. George's Hospital nursing staff has handed to the deputy-treasurer a draft for £2,046, the result of their sale of work and pound days held last autumn.

The East Sussex County Council has declined to co-operate in the establishment of a Local Committee for the county under the Voluntary Hospitals Commission.

Nearly half the £100,000 asked for by the Leicester Royal Infirmary has been received or promised. A remarkable recent gift was a cheque of £320 from the staff.

The salary of the matron of the Tiverton Isolation Hospital has been raised from £85 to £100. The present matron, Mrs. Wood, has been in the board's service for 20 years.

Aldeburgh has now a cottage hospital in the premises in the High Street, lately used for a Red Cross auxiliary hospital, and since as a private nursing home. Miss Petitt is hon. secretary.

Mr. W. B. Stoddart has been elected vice-chairman of the David Lewis Northern Hospital, Liverpool. From 1915 to 1920 he was honorary treasurer, and has been on the hospital committees since 1898.

A bed is to be endowed at St. Bartholomew's Hospital in memory of the late Sir Sydney Beauchamp, medical adviser to the British Delegation at the Peace Conference, who was trained at the hospital.

Dr. T. H. Somervell, of Brantfield, Kendal, is to accompany the Mount Everest Expedition. He is a painter, and will act as official artist to the expedition. He has done much climbing in the Lake District.

A novel collection, undertaken by the dairymen of the district, raised £325 for the Kensington and Fulham General Hospital, and thus averted, temporarily at any rate, the threatened closing of its wards.

The Sheffield Street Traders' Association, of which Mrs. Davies is the secretary, has adopted the penny-in-the-pound contribut ry scheme. The money is collected by weekly visits to the stallholders.

It is stated that the London hospitals now starting the provident scheme for the hospital treatment of subscribers hope to arrange an interchange of benefits with those provincial hospitals that have started kindred schemes.

An anonymous Italian waiter has given £1,000 to the Italian Hospital, "in remembrance of what has been done" for him and others there. A tablet thus inscribed is being placed over one of the beds to record this gift.

The value of bazaars organised by nurses is again apparent. The Newcastle Royal Victoria Infirmary has received £1,880 from the nursing staff through this means. By the nurses' wish £500 will be used to endow a cot at the infirmary.

Princess Mary has been asked by the Selby Urban Council if she will accept, in recognition of her marriage, the furnishing of a ward in the Selby and District War Memorial Hospital, about to be built, and consent to the ward being named after her.

Now that the price of butter has fallen to its pre-war level St. Bartholomew's Hospital has dispensed with margarine. A slight additional expense will be incurred, but the change is made in the interests of the health of the patients and nurses.

Lord Bearsted has presented an X-ray installation to the West Kent General Hospital. When the gift was handed over a speaker, on behalf of the hospital, said that a similar apparatus had been the means of restoring the donor to health.

The house adjoining the Thomas Knight Memorial Hospital, Blyth, is to be purchased and furnished as a nurses' home by the Blyth War Memorial Committee, which also proposes to furnish two memorial wards in the hospital for serious operations.

Dr. A. N. Robertson has been appointed Medical Superintendent of the Derbyshire Sanatorium. He has had institutional experience at Benenden and Sidlaw Sanatoria, and was previously County Tuberculosis Officer to Pembrokeshire.

Prince Henry has opened the Melton Mowbray War Memorial Hospital, which was presented to the district by Lt.-Col. Richard Dalgleish, who has converted the building at his own cost. The house, originally a hunting-box, stands in its own grounds of 15 acres.

The North Devon Infirmary has decided to require a contribution of 10s. a week from all patients able to pay; and by this means hope to meet the deficiency of £1,500 a year. This decision coincided with the appointment of Mr. G. F. Tregelles as honorary secretary.

Sir Walter Lawrence has resigned the chairmanship of the Council of King Edward VII. Sanatorium, Midhurst, and Sir Courtauld Thomson succeeds to the presidency. Sir Walter has been appointed vice-president to fill the vacancy created by the death of Sir Ernest Cassell.

The Holborn Division of the British Red Cross Society has presented the title deeds of Fernbank Hospital, Roehampton, and its site of nearly two acres, together with the balance remaining after its purchase and equipment, to the Ministry of Pensions. The hospital has been used for shell-shock cases.

Dr. Howard B. Cross, of the Rockefeller Institute, has died from yellow fever at Vera Cruz, where he had gone for the express purpose of combating the marsh and yellow fevers that curse that region, but within two days of arriving at Tuxtepec, in the course of his work, he contracted the disease. He was only 32 years of age.

The Mountain Ash Cottage Hospital, hitherto controlled by Lady Aberdare and a committee of colliery lodges and townspeople, has been handed over to the new hospital committee, of which Mr. T. Bowen is the secretary. The institution has been transferred at a nominal rent until the new hospital is ready. The road to the new site is being prepared by unemployed.

Professor B. Blacklock, Professor of the Tropical Diseases of Africa, together with Dr. Adler, both of the Liverpool School of Tropical Medicine, have now taken over the Sir Alfred Jones Research Laboratory recently opened at Freetown, Sierra Leone. Hitherto the activities of the school have been directed to sending out expeditions to tropical countries.

The King Edward VII. Welsh National Memorial Association is negotiating for the purchase of St. Bride's Castle, Pembrokeshire, with 150 acres. Alterations are to be deferred, but pavilions with open fronts will be built later. The Council disapproves of the suggestion from the Ministry of Health that charges for maintenance should be made on patients able to contribute.

The Lewisham Board of Guardians hopes to purchase Bromley Hill Court for a hospital. The house is historic for the meetings between William Pitt and Wilberforce which took place there during the anti-slavery agitation. The present owner of the house is Capt. de Valder, who had begun to change the building into flats. It was used as a convalescent home for Canadians during the war, and its present cost after alteration will be about £18,000. The house stands in 5 acres.

Ireland is supposed to be the home of quaint disputes. At Londonderry Infirmary the surgeon in charge lately resigned on the grounds that frivolous complaints and spiteful criticisms were made to him by the committee. He handed to the members the sum of eighteen pence, the cost of a melon, the purchase of which for him, he said, had evoked sarcasm, though it was the only melon bought for him during the previous eighteen months. The committee pressed him to remain in vain.

The Carnegie Heroes Fund Trustees has awarded a medallion and an annuity of £100 to Dr J. Hall Edwards, of Birmingham. It will be remembered that, as a result of X-ray experiments, Dr. Edwards contracted dermatitis, which necessitated the amputation of his left hand and forearm, and four fingers of the right hand. His disability began within a few months of the discovery of the X-rays, during which time he was experimenting night and day. He is still practising as a surgeon radiographer.

The Governors of St. Bartholomew's Hospital have accepted a tender for the building of the first wing of the Queen Mary's Home for St. Bartholomew's Nurses. The amount at present in hand (£113,000) includes a donation of £25,000 from Sir Edward Stern, in accordance with whose wishes the new wing will be associated with the name of the late Lady Stern. This portion of the home will accommodate 165 nurses, each of whom will have a separate bedroom. It is estimated that a total sum of £250,000 will be required to complete the home.

Sister M. Young has been elected a life governor of Norfolk and Norwich Hospital, in recognition of the £674 which during fifteen months ending last December she has collected from patients' visitors. The work, which was done in her own time, produced more than either the local house-to-house collection or the Rose Day. Mr. Charles Larking, the chairman, has recorded that the total collected by her is equivalent to nine times the sum which she has received in salary. She joined the staff in 1883. A resolution of thanks is to be inscribed on vellum and presented to Sister Young.

The Central Care Sub-Committee of the London Education Committee have made arrangements to provide for the medical and dental treatment of children attending their schools and institutions during the year beginning April 1, 1922, at a cost, apart from administrative charges, of £89,280. The sub-committee propose to establish six new centres under existing sanctions and four additional centres during 1922-23; four will be for the treatment of minor ailments and six for dental treatment. The number of cases for which it is proposed to make provision during 1922-23 is 226,458, an increase of 7,380.

Captain Sir Arthur Wellesley Clarke, K.B.E., J.P., an Elder Brother of Trinity House since 1898, has been elected chairman of the Seamen's Hospital Society (Dreadnought), in succession to the late Sir Perceval Alleyn Nairne. He is a member of the Port of London Authority and of the Council of the London Chamber of Commerce, and a Trinity Master, Admiralty Court. Captain H. B. Hooper, who has been appointed deputy-chairman, is well known in shipping circles in London, and has lately been appointed chairman of the sub-committees of classification of Lloyd's Register of British and Foreign Shipping.

The retirement of Mr. R. W. Harris, the Assistant Secretary in charge of the National Health Insurance Medical Benefits Department of the Ministry of Health, will be a serious loss to those interested in the work of insurance. All who have had to consult Mr. Harris regard him as a fair-minded Civil Servant with a keen humour and unfailing courtesy. He has been associated with national insurnce ever since the day that Mr. Lloyd George introduced his Bill in 1911, and is certainly one of the foremost experts on the question in the country. Mr. L. G. Brock, C.B., another Assistant Secretary, is taking the place of Mr. Harris.

